# Features of Luminescent Properties of Alginate Aerogels with Rare Earth Elements as Photoactive Cross-Linking Agents

**DOI:** 10.3390/gels8100617

**Published:** 2022-09-27

**Authors:** Vladislav Kaplin, Aleksandr Kopylov, Anastasiia Koryakovtseva, Nikita Minaev, Evgenii Epifanov, Aleksandr Gulin, Nadejda Aksenova, Peter Timashev, Anastasiia Kuryanova, Ilya Shershnev, Anna Solovieva

**Affiliations:** 1 N.N. Semenov Federal Research Center for Chemical Physics, Russian Academy of Sciences, 119991 Moscow, Russia; 2 Institute of Fine Chemical Technologies, Russian Technological University, 119571 Moscow, Russia; 3 Federal Research Centre “Crystallography and Photonics”, Institute of Photonic Technologies, Russian Academy of Sciences, Troitsk, 108840 Moscow, Russia; 4 Institute for Regenerative Medicine, I.M. Sechenov First Moscow State Medical University, 119991 Moscow, Russia; 5 Chemistry Department, Lomonosov Moscow State University, 119991 Moscow, Russia

**Keywords:** aerogels, lanthanide luminescence, supercritical carbon dioxide, sodium alginate, luminescent sensor

## Abstract

Luminescent aerogels based on sodium alginate cross-linked with ions of rare earth elements (Eu^3+^, Tb^3+^, Sm^3+^) and containing phenanthroline, thenoyltrifluoroacetone, dibenzoylmethane, and acetylacetone as ligands introduced into the matrix during the impregnation of alginate aerogels (AEG), were obtained for the first time in a supercritical carbon dioxide medium. The impregnation method used made it possible to introduce organically soluble sensitizing ligands into polysaccharide matrices over the entire thickness of the sample while maintaining the porous structure of the aerogel. It is shown that the pore size and their specific area are 150 nm and 270 m^2^/g, respectively. Moreover, metal ions with content of about 23 wt.%, acting as cross-linking agents, are uniformly distributed over the thickness of the sample. In addition, the effect of sensitizing ligands on the luminescence intensity of cross-linked aerogel matrices is considered. The interaction in the resulting metal/ligand systems is unique for each pair, which is confirmed by the detection of broad bands with individual positions in the luminescence excitation spectra of photoactive aerogels.

## 1. Introduction

The prospect of using rare earth elements (REE) in the creation of luminophores for analytical purposes, in particular for sensors, is usually associated not only with high quantum yields, long luminescence lifetimes, and a wide spectral range (from UV to IR), in which narrow-band luminescence of REE compounds is observed [1], but is determined by the possibility of regulating the functional characteristics of such systems when organic ligands of different natures are introduced (usually, the introduction of several ligands out of 4–6 possible options is conformationally acceptable) with the formation of specific photoactive centers “REE ion–ligands” [2,3,4]. Moreover, ligands also act as “antennas” [5,6,7] for radiation-initiating luminescence. This is even more important because of the low intensity of the luminescence of rare earth ions, caused by the forbidden electronic parity transitions [8]. The presence of third-party molecules or ions in the medium can affect the response of “antennas”. Thus, cellulose aerogels cross-linked with terbium and europium ions and exhibiting luminescence sensitive to K^+^, Ni^2+^, Co^2+^, Cu^2+^, and Fe^2+^ ions are described in [9]. Zhang et al. demonstrate the quenching of the luminescence of Eu^3+^ ions in a complex with YVO_4_ introduced into an alginate aerogel under the vapors of various organic solvents, including acetone, benzene, toluene, etc. [10]. Hai et al. show the binding of terbium ions with cellulose macromolecules using bridge ligands: 4-aminopyridine-2,6-dicarboxylic acid and 2-(2-aminobenzamido)benzoic acid. The resulting material exhibits reversible quenching/buildup of the luminescence of terbium ions in the presence of ClO^–^ and SCN^–^ ions, respectively [11].

The next range of problems that arise when creating analytical, in particular sensor, systems using luminescent complexes based on REE elements is associated with the formation of these complexes in polymer aerogels with a superporous cross-linked structure, bearing in mind the conformational possibilities for placement in such matrices of ions of organic ligands in the vicinity of REE. It is known [12] that for such purposes, the sodium salt of alginic acid can be chosen as the polymer base. Indeed, lanthanide ions, similar to calcium ions, are capable of binding with carboxyl groups of alginate polyanion macromolecules [13] to form three-dimensionally cross-linked gels, which acquire the structure of aerogels after treatment in a supercritical carbon dioxide (SC-CO_2_) medium [14]. The formation of three-dimensionally cross-linked structures prevents the extraction of ionically bound luminophore centers from the matrix during SC-CO_2_ drying [15], which occurs, for example, in lanthanide oxide aerogels [16]. At the same time, it should be noted that, although the methods for obtaining alginate aerogels lack such stages as hydrolysis, sol–gel formation, and sintering, which are characteristic of the synthesis of lanthanide-containing silicon and aluminum oxide aerogels [17], a certain problem associated with the search for common solvent for photoactive dopants and polymer matrix arises in the preparation of such aerogels. This problem is solved in this work based on a result previously obtained by the authors of the article. Thus, it is shown that organic sensitizing ligands, in particular phenanthroline, are easily introduced into cross-linked polymer matrices in a SC-CO_2_ medium [18]. By this method, for the first time, this work demonstrates the sensitization of the luminescence of rare earth ions in the composition of alginate aerogel matrices, performed in a supercritical CO_2_ medium. No similar researches were found in the literature. This made it possible to obtain luminescent aerogels based on sodium alginate cross-linked with lanthanide ions (Eu^3+^, Tb^3+^, Sm^3+^), and to establish the effects of the exposure of the formed systems of introduced sensitizing ligands (phenanthroline, dibenzoylmethane, thenoyltrifluoroacetone, acetylacetonate) on the luminescence intensity, with a possible perspective of using such systems as optical sensors for volatile organic substances.

## 2. Results and Discussion

### 2.1. Some Physicochemical Characteristics of Aerogel Matrices

#### 2.1.1. Specific Surface Area

**Figure 1:**
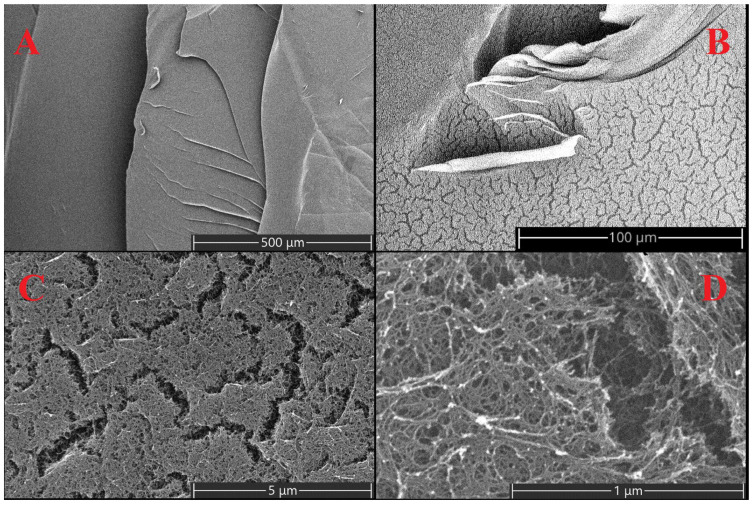
SEM images of the surface of an aerogel film cross-linked with Eu^3+^ ions: (**A**) magnification 100×, (**B**) 810×, (**C**) 15,000×, (**D**) 65,000×.

Superporous aerogel structures were obtained by drying in a supercritical CO_2_ medium (Figure 1). The average pore diameter is 149 nm ± 61 nm.

The data presented in Table 1 were obtained by the method of low-temperature adsorption of argon. The specific surface area (SSA) values of AEGs cross-linked by REE ions are comparable with similar values for some inorganic aerogels [19,20,21,22]. This makes it possible to use the obtained luminescent alginate aerogels as matrices in the development of sensors for the identification of gases and volatile substances.

#### 2.1.2. The Content of Rare Earth Metals in Cross-Linked Aerogel Matrices

**Figure 2:**
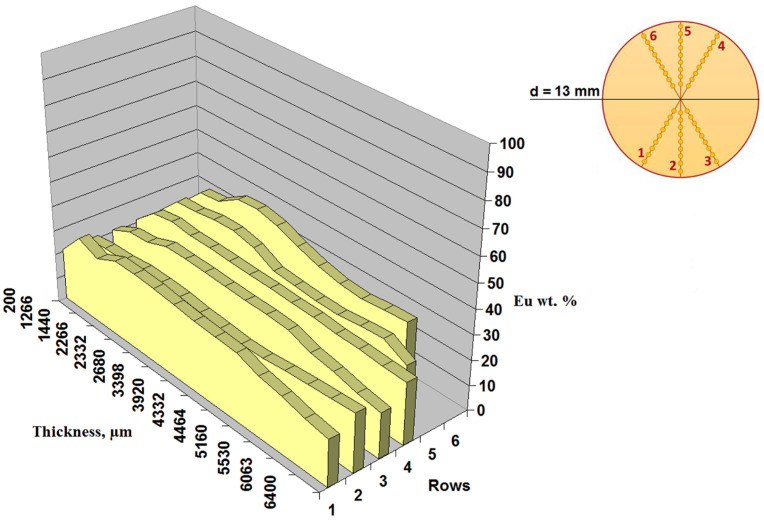
Mass content of Eu in a cross-linked matrix of sodium alginate.

Even after prolonged washing of the hydrogels, the metal content in the obtained aerogels remains constant, which indicates the fixation of all REE ions in the cross-linking sites of the alginate matrix. Therefore, the content of rare earth metals can provide information on the degree of cross-linking of the three-dimensional structure of the alginate aerogel. The elemental analysis data presented in Figure 2 as six data rows, correspond to six radial straight lines emerging from the center of the cylinder (200 µm) to its edges (6400 µm). The analysis was performed along each straight line at seven points. It can be seen that the mass content of europium ions is approximately the same over the entire cross-section of the Eu AEG sample (cylinder with a diameter of 13 mm), and is about 26%, which is close to the theoretical maximum content of europium (22.4 wt.%), corresponding to one trivalent europium ion per three carboxyl units. However, local measurements at the cut points lead to a high measurement error, about 23%. Therefore, the thermogravimetric method was used to determine the exact metal content in cross-linked aerogels.

The metal content was estimated by the thermogravimetric method, based on the fact that after thermal oxidation in air (at 1000 °C), the residue contains only metal oxide. The calculated maximum possible metal content (theoretical) and the content estimated using the thermogravimetric method (experimental) are presented in Table 2, and is also close to the EDS analysis data. The values that are lower than the theoretical ones are due to the incomplete reaction of the substitution of sodium ions by REE ions (about 85%), caused by steric hindrances in the formation of a cross-linked structure.

**Figure 3:**
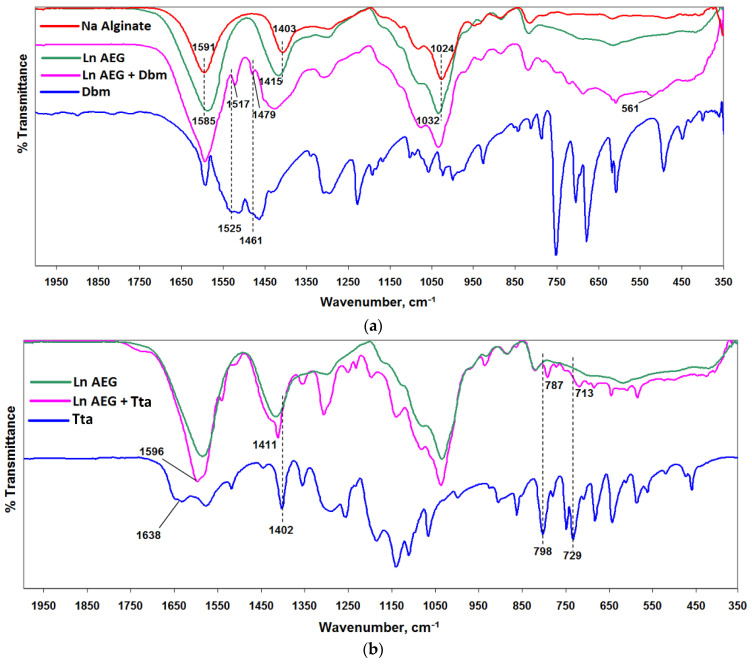

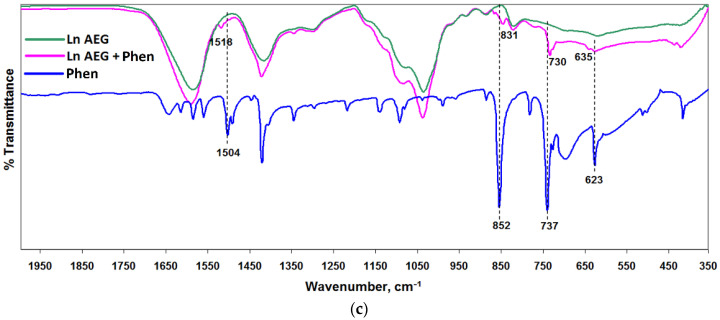
(**a**) FTIR spectra of sodium alginate, dibenzoylmethane, initial cross-linked films Ln AEG, and SC-impregnated films Ln AEG + Dbm. (**b**) FTIR spectra of thenoyltrifluoroacetone, initial cross-linked films Ln AEG, and SC-impregnated films Ln AEG + Tta. (**c**) FTIR spectra of phenanthroline, initial cross-linked films Ln AEG, and SC-impregnated films Ln AEG + Phen.

For additional characterization of the complexes formed upon SC impregnation of cross-linked aerogels with organic ligands, the materials were analyzed using FTIR. The FTIR spectra of the initial films and ligands, as well as the resulting systems, are shown in Figure 3a–c. As can be seen from the spectra, during the cross-linking of sodium alginate, the bands at 1403 cm^−1^ and 1591 cm^−1^ (characteristic bands of symmetric and antisymmetric C=O vibrations for salts of carboxylic acids) shift towards each other up to 1415 cm^−1^ and 1585 cm^−1^, respectively, when the Na^+^ ion is replaced by the lanthanide ion. The 1024 cm^−1^ band (C-O hydroxyl groups) also shifts to 1032 cm^−1^ under the influence of a more electronegative ion. The IR spectra of the impregnated films are a superposition of the most intense bands of the ligand on the spectrum of cross-linked alginate, with the exception of the Acac ligand, whose bands are not detected due to the extremely low concentration (Figure S1). According to other publications, the absorption bands of Dbm carbonyl groups do not undergo significant shifts relative to the absorption of the free ligand upon coordination with lanthanides [23]. For the films SC -impregnated with Dbm, shifts of the C=O vibration bands from 1525 cm^−1^ to 1517 cm^−1^ and C-H from 1461 cm^−1^ to 1479 cm^−1^ are observed (Figure 3b). In this case, a band at 516 cm^–1^ appears, which is related to the new Ln–O bond, and is absent in the initial ligand and film. Due to the low concentration of the ligand, this band is be detected in SC-impregnated films with the Tta ligand. However, it is known that the position of the C=O band of the Tta ligand shift down by about 40 cm^−1^ when combined with a rare earth ion [24,25]. For Ln AEG + Tta films, a shift of this band from 1638 cm^–1^ to 1596 cm^–1^ is observed, which confirms the coordination. On the spectra of films SC-impregnated with Phen, only a few of the most intense bands belonging to phenanthroline are detected. However, their position is also shifted relative to the bands of free phenanthroline, which is typical for Phen complexes with metal: from 623 cm^−1^ to 635 cm^−1^, from 737 cm^−1^ to 730 cm^−1^, from 852 cm^−1^ to 841 cm^−1^, and from 1504 cm^−1^ to 1518 cm^−1^ (Figure 3c).

### 2.2. Effect of Organic Sensitizing Ligands on the Luminescent Properties of Aerogel Polysaccharide Matrices Cross-Linked with REE Ions

#### 2.2.1. Luminescence of Aerogel Films

Well-studied ligands with known triplet energy levels close to the radiative levels of a given series of metals, as well as well-soluble in SC-CO_2_ medium, were used for sensitization of luminescence: acetylacetone, phenanthroline, dibenzoylmethane, and thenoyltrifluoroacetone. Based on the difference between the energies of the triplet level of the ligand and the radiative level of the metal, which should be in the range of 1000 cm^–1^–5500 cm^–1^ [26], one can predict in advance the efficiency of energy transfer from the ligand to the metal [27,28,29,30]. Table 3 lists the energies of the triplet levels of the abovementioned ligands and the radiative levels of Eu^3+^, Tb^3+^, and Sm^3+^ ions, as well as the difference between these energies (ΔE) for each metal/ligand pair. Green color indicates the pairs for which an effective sensitization process is expected and, as a result, an increase in the intensity of the luminescence of rare earth ions. Highlighted in red are ΔE, at which the energy transfer from the ligand to the metal either does not occur (ΔE > 5500 cm^−1^), or at which the reverse energy transfer dominates (ΔE < 1500 cm^−1^).

**Figure 4:**
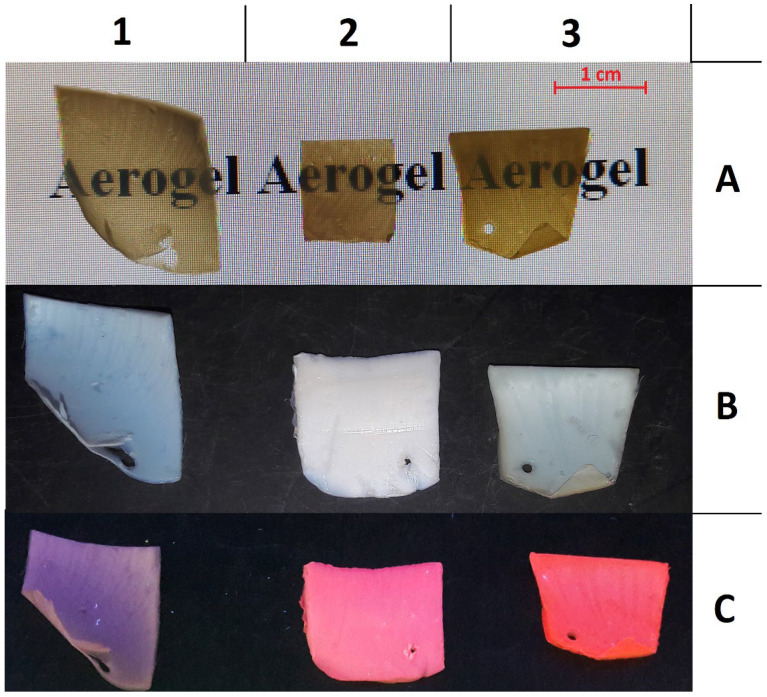
Eu AEG (**1**), Eu AEG + Phen (**2**), and Eu AEG + Tta (**3**) aerogels in transmitted light (row **A**), daylight (row **B**), and under 365 nm UV light (row **C**).

Thus, the effective REE–ligand interaction should be observed for Eu and Sm systems with Tta, Phen, and Dbm, and for Tb with Phen and Acac. The efficiency of the sensitization process after the SC introduction of ligands was evaluated by the increase in the luminescence intensity of aerogel matrices cross-linked with rare earth ions, and the occurrence of metal–ligand interaction by changes in the luminescence excitation spectra. Indeed, the change in the luminescence intensity for all AEGs occurs in accordance with the expected results, with the exception of the Eu AEG + Dbm pair. Dbm molecules show a weaker sensitizing ability compared to other ligands, and no sensitization is observed in the Eu AEG matrix. This can be explained by the features of the keto–enol equilibrium of the Dbm tautomers in the nonpolar SC-CO_2_ medium [31,32]. In the luminescence spectra of aerogel matrices cross-linked with Eu^3+^ and Tb^3+^ ions, characteristic narrow bands of low intensity metal-centered luminescence are observed. Moreover, the luminescence excitation spectra are also represented by a set of narrow bands, the positions of which are given in Table 4. At the same time, the characteristic luminescence (bands at 563 nm, 598 nm, and 644 nm) are not detected in the samples cross-linked with Sm^3+^ ions. The excitation and luminescence spectra of cross-linked aerogels are presented in Figures S2 and S3. Before SC impregnation, the samples are transparent white or slightly yellow. After the introduction of ligands in the SC-CO_2_ medium, the transparency is preserved, and the films acquire a pink (for Phen and Dbm) or yellow (for Acac and Tta) tint. Figure 4 shows the films of the original Eu AEG (1) and of the Eu AEG SC-impregnated with Phen (2) and Tta (3) ligands. In the first row (A), the films are placed on a light monitor. Transparency is also confirmed by the absorption spectra of the original Eu AEG film and of the Eu AEG impregnated with Phen ligands (Figure S4). The second row (B) shows the films in daylight, the third row (C) shows the films exposed to 365 nm UV light. The remaining samples have a similar appearance, except that their glow under the ultraviolet light is not apparent to the naked eye.

In all cases, after SC impregnation of the matrices, the excitation spectra are represented by broad bands (from 140 to 240 nm wide) that are characteristic for organic molecules (Table 4). Correspondingly, ligands dissolved in SC fluid are adsorbed on the surface and in the volume of aerogels, and coordinate near REE ions, forming luminescent systems with them. All recorded spectra of the SC-impregnated aerogels are shown in Figures S5–S15.

In Table 4, green indicates an increase in luminescence intensity after impregnation with organic ligands, while red indicates a decrease in intensity.

It is known that the standard procedure for obtaining luminescent organic REE complexes includes mixing solutions of metal salts and ligands, adjusting pH to a certain value, and isolating and purifying the precipitated product [33]. The obtained complexes are no longer able to act as cross-linking agents for water-soluble polyanions, since they become insoluble in aqueous media. On the other hand, the introduction of a ready-made luminescent REE complex into cross-linked aerogels (for example, by impregnation in SC-CO_2_) is limited by solubility in SC-CO_2_, concentration quenching, and aggregation. For alginate aerogels cross-linked with REE, each luminescent center is located in the cross-link site and is shielded from neighboring centers by fragments of polymer molecules. Therefore, the sensitization of such distributed luminescent centers in alginate matrices can be considered as a way to bypass the problem of concentration quenching. However, luminescence quenching is observed in aerogels with an ion content of about 20%. Thus, the maximum luminescence intensity is achieved at a REE content of about 10 wt.%. Also, preliminary tests of the luminescence sensitivity of some aerogel matrices to the presence of organic and inorganic vapors (acetone, ammonia) were carried out. The original non-impregnated aerogels cross-linked with REE are not sensitive to the tested volatile compounds. It is interesting to note that only one matrix (AEG Eu, containing Tta) shows the reaction to acetone after SC impregnation: the intensity of characteristic luminescence bands in the presence of acetone vapor increase by 32% (Figure S16). Luminescence quenching by ammonia vapor is observed in AEG Eu and AEG Tb aerogels SC-impregnated with Phen. The luminescence intensity of the matrices drops by 18% and 60%, respectively (Figures S17 and S18). Thus, not only the structure of the organic ligand, but also the metal, has a significant effect on the nature of the response of luminescent aerogels.

#### 2.2.2. Features of the Distribution of Impregnated Ligands in the Volume of Aerogels

**Figure 5:**
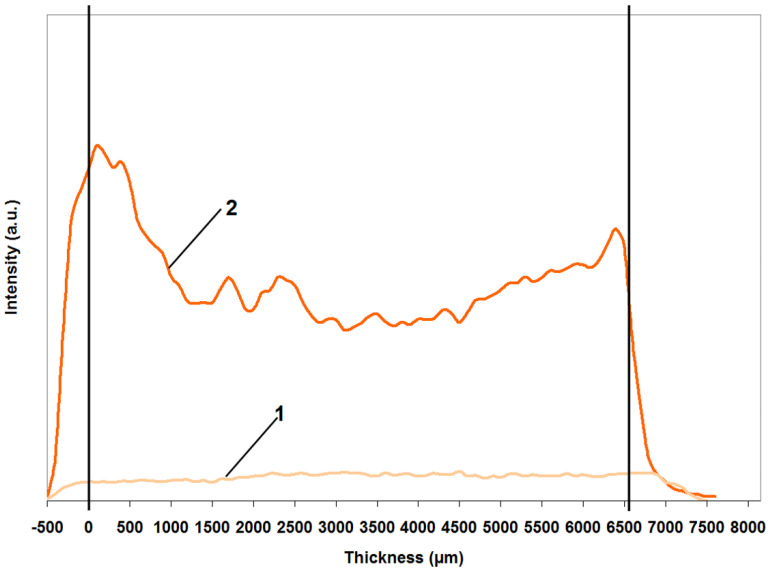
Distribution of luminescence intensity at 613 nm over a section of the Eu AEG–Phen sample: (1) luminescence intensity in the initial matrix, (2) intensity after impregnation with phenanthroline.

It is important to note that when mentioning the most characteristic properties of aerogel materials, such as porosity, mechanical properties, and refractive index, three-dimensional structure properties are implied. This also applies to luminescent properties: up to a certain thickness, aerogels are optically transparent for the UV–NIR range; accordingly, radiation should occur not only from rare earth ions localized on the surface of the matrix, but also in its volume. Therefore, it is necessary to make sure that the interaction of luminescent ions with the introduced sensitizing ligands occurs through the volume of the matrix. For example, Zhang et al. demonstrate the penetration of a sensitizing ligand into a sample layer no thicker than 130 μm, after impregnating an aerogel matrix in solution [10]. This work shows that the impregnation of alginate aerogels in the SC-CO_2_ medium ensures the impregnation of the matrix to a depth of at least 3.3 mm (cylinder with a radius of 6.6 mm) (Figure 5). At the same time, this value is still limited only by the difficulties in obtaining thicker aerogel blocks, but not by the capabilities of the SC-fluid. Figure 5 shows the intensity distribution of the luminescence signal at 613 nm over the thickness of the Eu AEG (curve 1) and Eu AEG–Phen (after SC impregnation) matrices (curve 2). It can be seen that the increase in signal intensity occurs throughout the entire volume of the matrix, which indicates the penetration of the ligand dissolved in the SC-medium to a given depth. The increased values from the edges of the matrix (0–100 µm, 6000–6500 µm) are associated with a more intense diffusion of the solution into the near-surface layers.

## 3. Conclusions

The luminescent aerogels based on sodium alginate, cross-linked with ions of rare earth elements (Eu^3+^, Tb^3+^, Sm^3+^) and containing phenanthroline, thenoyltrifluoroacetone, dibenzoylmethane, and acetylacetonate as ligands, introduced into the matrix during SC impregnation of alginate aerogels, were obtained in a supercritical carbon dioxide medium for the first time. It is shown that the intensity of the luminescence bands change after impregnation. Moreover, the nature of the influence of organic additives (ligands) on the luminescent properties of REE ions depends on the nature of both the ion and the ligand. It is demonstrated that upon SC impregnation, ligands can penetrate and act as luminescence sensitizers of rare earth ions throughout the entire thickness of aerogels.

## 4. Materials and Methods

### 4.1. Preparation of Alginate Aerogels Cross-Linked with REE Ions

The following substances were used without additional preparation and purification: REE chloride hexahydrates: XCl_3_x6H_2_O, where X is Eu, Tb, Sm (Aldrich, St. Louis, MO, USA, 99.9%); gadolinium (III) acetylacetonate hydrate (Gd(Acac)_3_xH_2_O) (Aldrich, 99.9%); europium (III) theonyltrifluoroacetonate trihydrate (Eu(Tta)_3_x3H_2_O) (Acros Organics, Geel, Belgium 95%); sodium alginate (Rushim, Moscow, Russia); sensitizing ligands: 1,10-phenanthroline (Acros Organics, 99+%); thenoyltrifluoroacetone (Aldrich, 99+%); dibenzoylmethane (Aldrich, 99+%); isopropanol (HIMFARM, Moscow, Russia, TU 2632-181-44493179-2014) (hereinafter, coordination water is not indicated for REE compounds).

To create supercritical conditions for impregnation and drying, dry carbon dioxide, with the volume content of water vapor not exceeding 0.001%, according to the quality certificate, was utilized (OOO “NII KM” 99.8% All-Union State Standard 8050-85).

Alginate aerogels in the form of films and cylinders were obtained by the following method. First, hydrogel films were obtained by pouring 40 mL of an aqueous solution of REE chloride (5 wt.%) into 30 mL of an aqueous solution of sodium alginate (2 wt.%) in a plastic Petri dish (d = 85 mm). The thickness of the formed film varied from 1 mm along the edges to 3 mm in the center. Hydrogel cylinders of 2 cm in diameter were obtained by squeezing 10 mL of a 2% aqueous solution of sodium alginate into a 10-fold excess of a 5% aqueous solution of REE chloride from a 10 mL syringe. The hydrogels were kept in distilled water for 72 h, changing the water three times to remove unreacted REE chloride. Then, the water in the hydrogels was replaced with isopropanol: the hydrogels were kept in a mixture of isopropanol/water (25/75) for 24 h, and then the proportion of isopropanol was increased by 25% once a day, bringing it to 100%.

**Figure 6:**
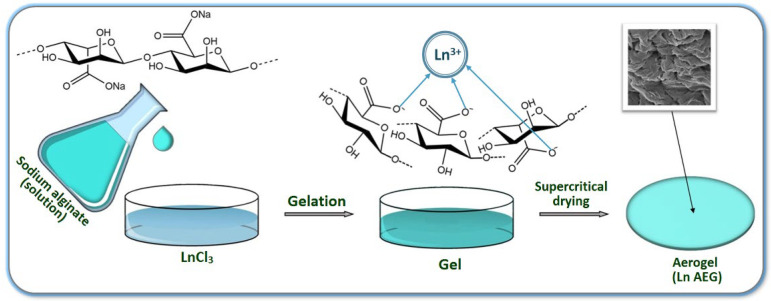
Diagram for obtaining aerogel films cross-linked with REE ions.

The cross-linked alginate hydrogels were dried in a high-pressure flow reactor in supercritical carbon dioxide at a temperature of 40 °C and a pressure of 115 bar. The diagram of the process is shown in Figure 6.

### 4.2. Impregnation of Aerogels Cross-Linked with REE Ions by the Organic Ligands

**Figure 7:**
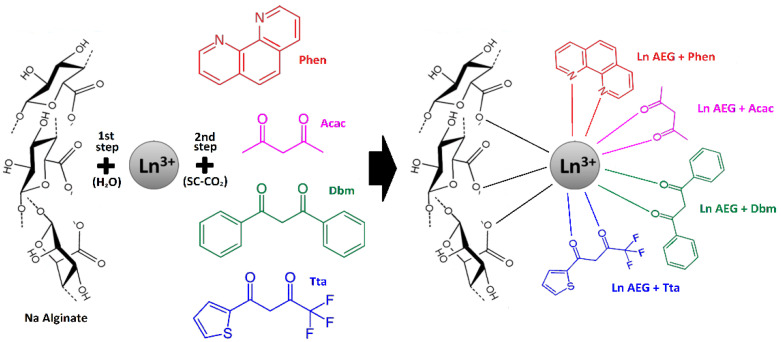
Diagram of complex formation of new systems of REE-containing alginate and sensitizing ligands Tta, Phen, Acac, and Dbm.

Aerogels were impregnated with the organic ligands in SC-CO_2_ medium. The concentration of ligands in the supercritical solution was 0.25 mg/mL. The impregnation was carried out for 1 h at a pressure of 180 bar and a temperature of 90 °C. Previously, in our work it is shown that, under these conditions, it is possible to achieve a uniform distribution of impregnated compounds in various polymer matrices in a SC-CO_2_ medium [34]. The reactor was then cooled to room temperature and depressurized to atmospheric pressure for 30 min (Figure 7).

### 4.3. Determination of Luminescent and Physicochemical Characteristics of Cross-Linked Aerogel Matrices

The luminescence and luminescence excitation spectra of the aerogel films were recorded using a Horiba Fluoromax Plus (Horiba-Jobin-Yvon, Palaiseau, France) spectrofluorometer at room temperature. The distribution of the luminescence intensity over the thickness of the aerogel cylinders was determined using a flexible optical fiber with a diameter of 0.8 mm directed at the cross-section of the sample, and a QE Pro 65000 spectrometer (Ocean Insight, Orlando, FL, USA). The displacement was provided by a movable stage with a positioning accuracy of 10 ± 1 μm. The values were recorded from the surface of the cross-section of the cylinder along a straight line from the periphery to the center with a step of 100 µm.

The specific surface area (SSA) of polysaccharide aerogels was determined by the low-temperature argon adsorption method (BET method). The analysis was carried out at the V.V. Voevodsky Laboratory of Kinetics of Mechanochemical and Free-Radical Processes (N.N. Semenov Federal Research Center for Chemical Physics, RAS, Moscow, Russia).

SEM images of the porous structure of aerogels were obtained using a scanning electron microscope Prisma E (Thermo Fisher Scientific, Scheepsbouwersweg, The Netherlands) after deposition of a layer of gold (10 nm). Data on the metal content in aerogel matrices were obtained from the surface of a cross-section of a cylindrical sample using a Phenom ProX scanning electron microscope (Thermo Fisher Scientific, Scheepsbouwersweg, The Netherlands) equipped with an energy-dispersive spectroscopy (EDS) silicon drift detector, which allows the performance of elemental analysis. Also, the metal content in the matrices was determined by the gravimetric method, based on the residue after burning the samples in a Saturn 1 high-temperature furnace at a temperature of 1000 °C.

FTIR analysis of the initial components and the synthesized system was carried out using a spectrum two FT-IR spectrometer (PerkinElmer, Waltham, MA, USA) in attenuated total reflectance (ATR) mode. The spectrometer features were as follows: high-performance, room-temperature LiTaO_3_ MIR detector, standard optical system with KBr windows for data collection over a spectral range of 4000–350 cm^−1^ at a resolution of 0.5 cm^−1^. All spectra were initially collected in ATR mode and converted into IR transmittance mode.

## Figures and Tables

**Table 1:** Specific surface area of alginate aerogel films cross-linked with REE ions.

Aerogel Matrix	SSA Average Value, m^2^/g
Eu AEG	255 ± 22
Tb AEG	290 ± 43
Sm AEG	270 ± 9
Inorganic AEG	≈100–2300

**Table 2:** Experimental and theoretical mass content of metal in alginate aerogel films cross-linked with Eu^3+^, Tb^3+^, and Sm^3+^ ions.

Sample	Metal Content (Experimental), wt.%	Metal Content (Theoretical), wt.%
Eu AEG	19.4 ± 0.3	22.4
Tb AEG	20.9 ± 0.2	23.2
Sm AEG	17.9 ± 0.2	22.2

**Table 3:** The energies of the triplet levels of the Tta, Phen, Acac, and Dbm ligands, the energies of the excited radiative levels of the Eu^3+^, Tb^3+^, and Sm^3+^ ions, and their difference ΔE for each metal/ligand pair.

	Tta: 20,500 cm^−1^	Phen: 22,075 cm^−1^	Acac: 25,310 cm^−1^	Dbm: 20,300 cm^−1^
Eu (^5^D_0_) (17,267 cm^−1^)	3233 cm^−1^	4808 cm^−1^	8043 cm^−1^	3033 cm^−1^
Tb (^5^D_4_) (20,394 cm^−1^)	106 cm^−1^	1681 cm^−1^	4916 cm^−1^	−94 cm^−1^
Sm (^4^G_5/2_) (17,825 cm^−1^)	2675 cm^−1^	4250 cm^−1^	7485 cm^−1^	2475 cm^−1^

**Table 4:** The position of the maxima of the original alginate aerogels cross-linked with REE ions and aerogels after SC impregnation with organic ligands, as well as changes in the luminescence intensity. The letters “S”, “M”, and “W” denote strong, medium, and weak luminescence bands, respectively.

Sample	Position of Band Maxima in the Luminescence Excitation Spectra	Changes in the Intensity of Characteristic Luminescence Peaks of REE Ions
**Initial alginate aerogels cross-linked with REE ions**		
Eu AEG	393 nm; 464 nm	577, 590, 615 (S)
Tb AEG	317 nm; 340 nm; 351 nm; 368 nm; 377 nm	488, 543, 584, 620 (M)
Sm AEG	**--**	563, 598, 644 (Not detected)
**Matrices impregnated with thenoyltrifluoroacetone (Tta)**		
Eu AEG +Tta	363 nm	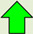 (S)
Tb AEG +Tta	290 nm; 356 nm; 410 nm	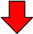 (W)
Sm AEG +Tta	368 nm	Luminescence (M)
**Matrices impregnated with phenanthroline (Phen)**		
Eu AEG +Phen	350 nm	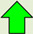 (S)
Tb AEG +Phen	347 nm	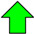 (S)
Sm AEG +Phen	368 nm	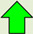 (M)
**Matrices impregnated with acetylacetone (Acac)**		
Eu AEG +Acac	338 nm	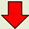 (M)
Tb AEG +Acac	304 nm	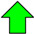 (S)
Sm AEG +Acac	**--**	No luminescence
**Matrices impregnated with dibenzoylmethane (Dbm)**		
Eu AEG +Dbm	386 nm	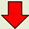 (W)
Tb AEG +Dbm	295 nm	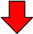 (W)
Sm AEG +Dbm	395 nm	Luminescence (W)

## Data Availability

Data are contained within the article or supplementary material.

## Supplementary Materials

The following supporting information can be downloaded at: https://www.mdpi.com/article/10.3390/gels8100617/s1, Figure S1: FTIR spectra of initial cross-linked films Ln AEG and SC-impregnated films Ln AEG + Acac; Figures S2 and S3: Luminescence spectra (orange curve) and luminescence excitation spectra (green curve) of alginate aerogels cross-linked with Eu^3+^ and Tb^3+^ ions, respectively; Figure S4: Absorption spectra of Eu AEG (curve 1) and Eu AEG film after SC impregnation of Phen (curve 2). Figures S5–S15: Luminescence (orange curve) and luminescence excitation (green curve) spectra of alginate aerogels cross-linked with Eu^3+^, Tb^3+^, and Sm^3+^ ions, SC-impregnated with Tta, Phen, Acac, and Dbm ligands; Figure S16: Luminescence spectra: 1.AEG Eu SC-impregnated with Tta before and 2. after exposure to acetone vapor; Figure S17: Luminescence spectra: 1. AEG Eu SC-impregnated with Phen before and 2. after exposure to ammonia vapor, Figure S18: Luminescence spectra: 1. AEG Tb SC-impregnated with Phen before and 2. after exposure to ammonia vapor.

## References

[1] Huang C.-H. (2010). Rare Earth Coordination Chemistry: Fundamentals and Applications.

[2] Liu J., Liang Q.-B., Wu H.-B. (2016). Synthesis, photophysics, electrochemistry, thermal stability and electroluminescent performances of a new europium complex with bis(β-diketone) ligand containing carbazole group: Luminescent Performances of a New Europium Complex. Luminescence.

[3] Turchetti D.A., Domingues R.A., Zanlorenzi C., Nowacki B., Atvars T.D.Z., Akcelrud L.C. (2014). A Photophysical Interpretation of the Thermochromism of a Polyfluorene Derivative–Europium Complex. J. Phys. Chem. C.

[4] Turchetti D.A., Nolasco M.M., Szczerbowski D., Carlos L.D., Akcelrud L.C. (2015). Light emission of a polyfluorene derivative containing complexed europium ions. Phys. Chem. Chem. Phys..

[5] George M.R., Critchley P.E., Whitehead G.F., Bailey A.J., Cuda F., Murdin B.N., Grossel M.C., Curry R.J. (2021). Modified pyridine-2,6-dicarboxylate acid ligands for sensitization of near-infrared luminescence from lanthanide ions (Ln3+ = Pr3+, Nd3+, Gd3+, Dy3+, Er3+). J. Lumin.

[6] Wang S., Chu X., Xiang X., Cao Y. (2019). Highly selective antenna effect of graphene quantum dots (GQDs): A new fluorescent sensitizer for rare earth element terbium in aqueous media. Talanta.

[7] Sun N.-N., Yan B. (2018). Near-infrared emission sensitization of lanthanide cation based on Ag+ functionalized metal-organic frameworks. J. Alloy. Compd..

[8] Sabbatini N., Guardigli M., Lehn J.-M. (1993). Luminescent lanthanide complexes as photochemical supramolecular devices. Coord. Chem. Rev..

[9] Fan W., Du J., Kou J., Zhang Z., Liu F. (2018). Hierarchical porous cellulose/lanthanide hybrid materials as luminescent sensor. J. Rare Earths.

[10] Zhang Z.-Y., Zhu H., Xu Q.-Q., Liu F.-Y., Zhu A.-X., Kou J.-F. (2019). Hybrid luminescent alginate hydrogels containing lanthanide with potential for acetone sensing. New J. Chem..

[11] Hai J., Li T., Su J., Liu W., Ju Y., Wang B., Hou Y., Liu W. (2018). Reversible Response of Luminescent Terbium(III)-Nanocellulose Hydrogels to Anions for Latent Fingerprint Detection and Encryption. Angew. Chem..

[12] Zhang Z., Liu F., Xu Q., Zhu H., Zhu A., Kou J. (2019). Covalent Grafting Terbium Complex to Alginate Hydrogels and Their Application in Fe^3+^ and pH Sensing. Glob. Challenges.

[13] Liu F., Carlos L.D., Ferreira R.A.S., Rocha J., Gaudino M.C., Robitzer M., Quignard F. (2008). Photoluminescent Porous Alginate Hybrid Materials Containing Lanthanide Ions. Biomacromolecules.

[14] Robitzer M., David L., Rochas C., Di Renzo F., Quignard F. (2008). Nanostructure of Calcium Alginate Aerogels Obtained from Multistep Solvent Exchange Route. Langmuir.

[15] Sorensen L., Strouse G.F., Stiegman A.E. (2006). Fabrication of Stable Low-Density Silica Aerogels Containing Luminescent ZnS Capped CdSe Quantum Dots. Adv. Mater..

[16] Tillotson T.M., Sunderland W.E., Thomas I.M., Hrubesh L.W. (1994). Synthesis of lanthanide and lanthanide-silicate aerogels. J. Sol.-Gel Sci. Technol..

[17] Małecka M.A., Kępiński L. (2012). Solid state reactions in highly dispersed single and mixed lanthanide oxide–SiO_2_ systems. Catal. Today.

[18] Kaplin V.S., Kopylov A.S., Zarhina T.S., Timashev P.S., Solov’Eva A.B. (2020). Luminescent Properties of Mixed-Ligand Neodymium β-Diketonates Obtained in Supercritical Carbon Dioxide in Polymer Matrices of Various Nature. Opt. Spectrosc..

[19] Alwin S., Ramasubbu V., Shajan X.S. (2018). TiO_2_ aerogel–metal organic framework nanocomposite: A new class of photoanode material for dye-sensitized solar cell applications. Bull. Mater. Sci..

[20] Nguyen B.N., Meador M.A.B., Scheiman D., McCorkle L. (2017). Polyimide Aerogels Using Triisocyanate as Cross-linker. ACS Appl. Mater. Interfaces.

[21] Maleki H., Durães L., Portugal A. (2014). An overview on silica aerogels synthesis and different mechanical reinforcing strategies. J. Non-Crystalline Solids.

[22] Li H., Li J., Thomas A., Liao Y. (2019). Ultra-High Surface Area Nitrogen-Doped Carbon Aerogels Derived From a Schiff-Base Porous Organic Polymer Aerogel for CO_2_ Storage and Supercapacitors. Adv. Funct. Mater..

[23] Kanimozhi A.J., Alexander V. (2017). Synthesis and photophysical and magnetic studies of ternary lanthanide(iii) complexes of naphthyl chromophore functionalized imidazo[4,5-f][1,10]phenanthroline and dibenzoylmethane. Dalton Trans..

[24] Wang Y.-P., Luo Y., Wang R.-M., Yuan L. (1997). Synthesis and fluorescence properties of the mixed complexes of Eu(III) with polymer ligand and thenoyl trifluoroacetone. J. Appl. Polym. Sci..

[25] Wang L.-H., Wang W., Zhang W.-G., Kang E.-T., Huang W. (2000). Synthesis and Luminescence Properties of Novel Eu-Containing Copolymers Consisting of Eu(III)−Acrylate−β-Diketonate Complex Monomers and Methyl Methacrylate. Chem. Mater..

[26] Xu H., Sun Q., An Z., Wei Y., Liu X. (2015). Electroluminescence from europium(III) complexes. Co-ord. Chem. Rev..

[27] Freidzon A.Y., Kurbatov I.A., Vovna V.I. (2018). *Ab initio* calculation of energy levels of trivalent lanthanide ions. Phys. Chem. Chem. Phys..

[28] Smirnova T.D., Shtykov S.N., Kochubei V.I., Khryachkova E.S. (2011). Excitation energy transfer in europium chelate with doxycycline in the presence of a second ligand in micellar solutions of nonionic surfactants. Opt. Spectrosc..

[29] Jinghe Y., Xuezhen R., Huabin Z., Ruiping S. (1990). Enhanced luminescence of the europium(III)-terbium(III)-dibenzoylmethane-ammonia-acetone system and its application to the determination of europium. Analyst.

[30] Gusev A.N., Hasegawa M., Shimizu T., Fukawa T., Sakurai S., Nishchymenko G.A., Shul’Gin V.F., Meshkova S.B., Linert W. (2013). Synthesis, structure and luminescence studies of Eu(III), Tb(III), Sm(III), Dy(III) cationic complexes with acetylacetone and bis(5-(pyridine-2-yl)-1,2,4-triazol-3-yl)propane. Inorganica Chim. Acta.

[31] Kojić M., Lyskov I., Milovanović B., Marian C.M., Etinski M. (2019). The UVA response of enolic dibenzoylmethane: Beyond the static approach. Photochem. Photobiol. Sci..

[32] AL-Hilfi J.A. A Structural Study of 2-Thenoyltrifluoroacetone Schiff Bases and Their Thione Derivatives: Synthesis, NMR and IR. AIP Conference Proceedings. Proceedings of the 8th International Conference on Applied Science and Technology (ICAST 2020).

[33] Ugale A., Kalyani T.N., Dhoble S.J. (2018). Potential of Europium and Samarium β-Diketonates as Red Light Emitters in Organic Light-Emitting Diodes. Lanthanide-Based Multifunctional Materials.

[34] Kopylov A.S., Yusupov V.I., Cherkasova A.V., Shershnev I.V., Timashev P.S., Solovieva A.B. (2018). The Distribution Features of Photoactive Fillers in Different-Nature Polymer Matrices upon Their Impregnation in a Supercritical Carbon Dioxide Medium. Russ. J. Phys. Chem. B.

