# Supplementary material for: Features of Luminescent Properties of Alginate Aerogels with Rare Earth Elements as Photoactive Cross-Linking Agents
Source: Gels. 2022 Sep 27;8(10):617. doi: 10.3390/gels8100617 (PMC9602161; doi:10.3390/gels8100617)
Supplement: Supplementary file 1 [file gels-08-00617-s001.zip › gels-1896316-supplementary.pdf]

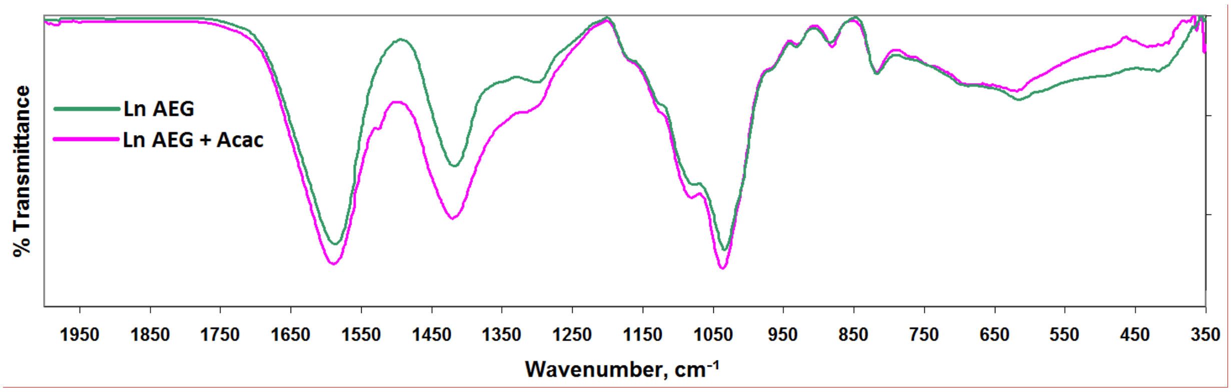


Figure S1. FTIR spectra of initial crosslinked films Ln AEG and SC-impregnated films Ln AEG + Acac.

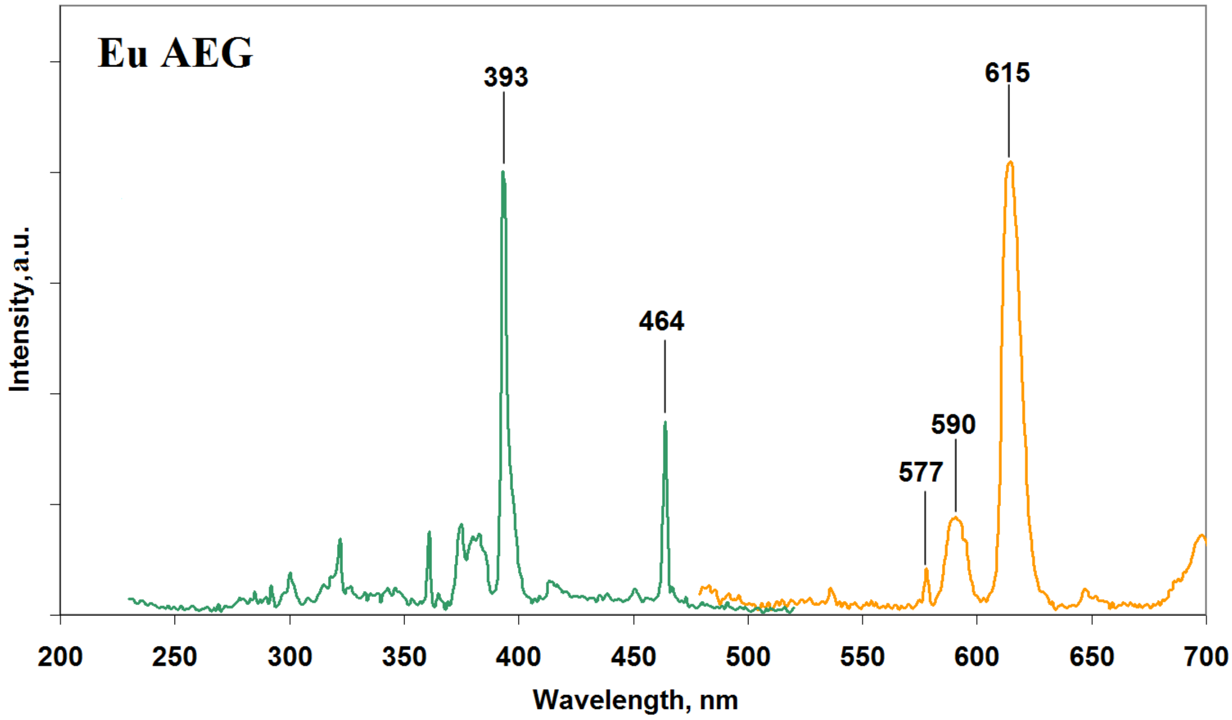


Figure S2. Luminescence spectra (orange curve) and luminescence excitation spectra (green curve) of alginate aerogels crosslinked with $Eu^{3+}$.

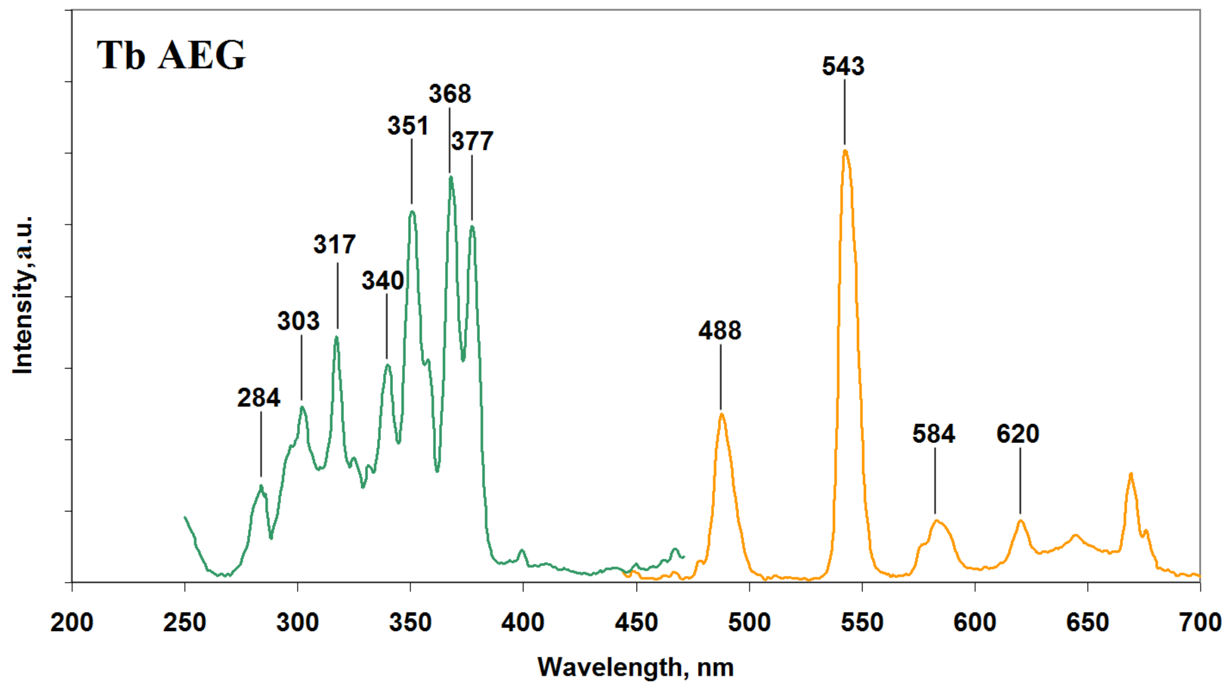


Figure S3. Luminescence spectra (orange curve) and luminescence excitation spectra (green curve) of alginate aerogels crosslinked with $Tb^{3+}$.

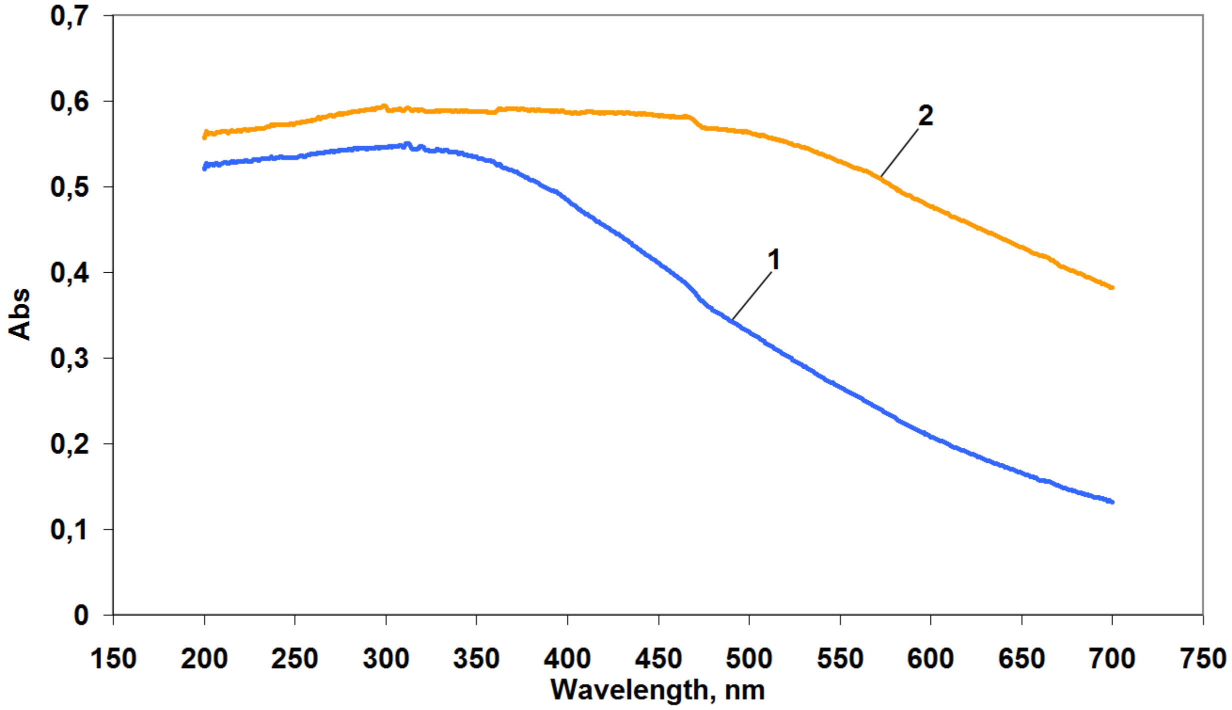


Figure S4. Spectra of Eu AEG (curve 1) and Eu AEG film after SC-impregnation of Phen (curve 2). Film thickness approximately 0,3 мм.

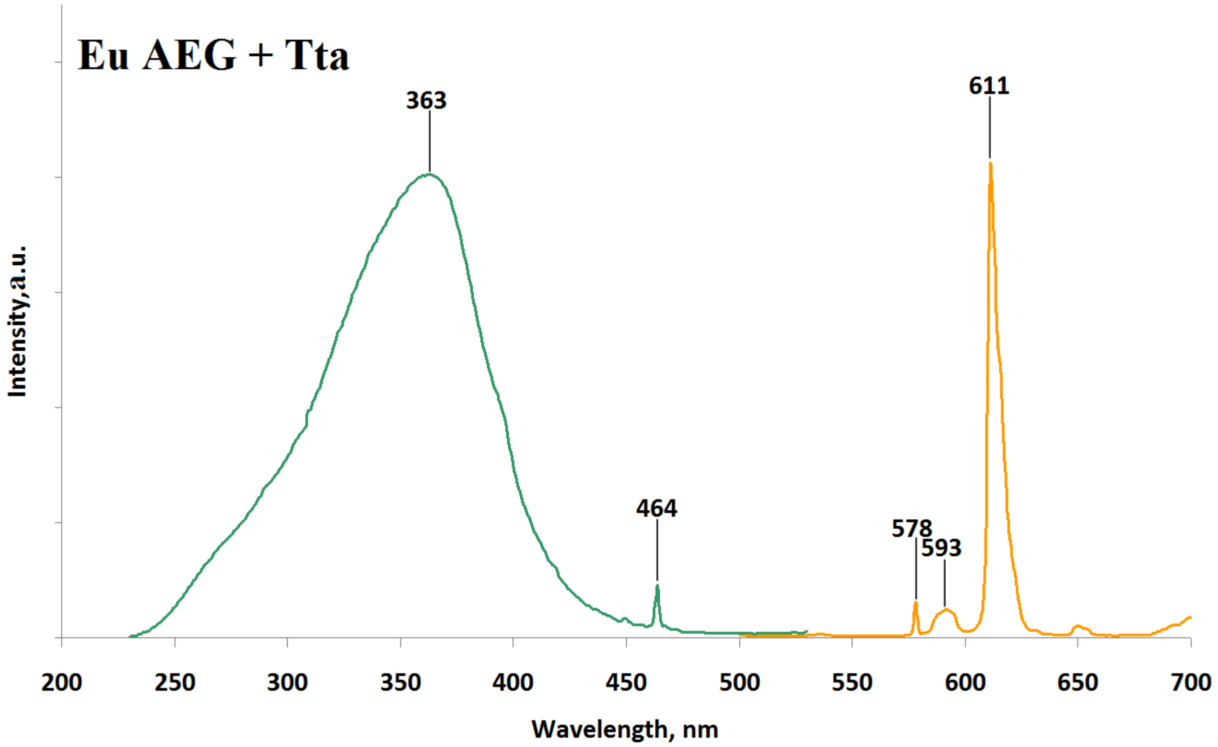


Figure S5. Luminescence (orange curve) and luminescence excitation (green curve) spectra of alginate aerogels crosslinked with $Eu^{3+}$ ions, SC-impregnated with Tta ligands.

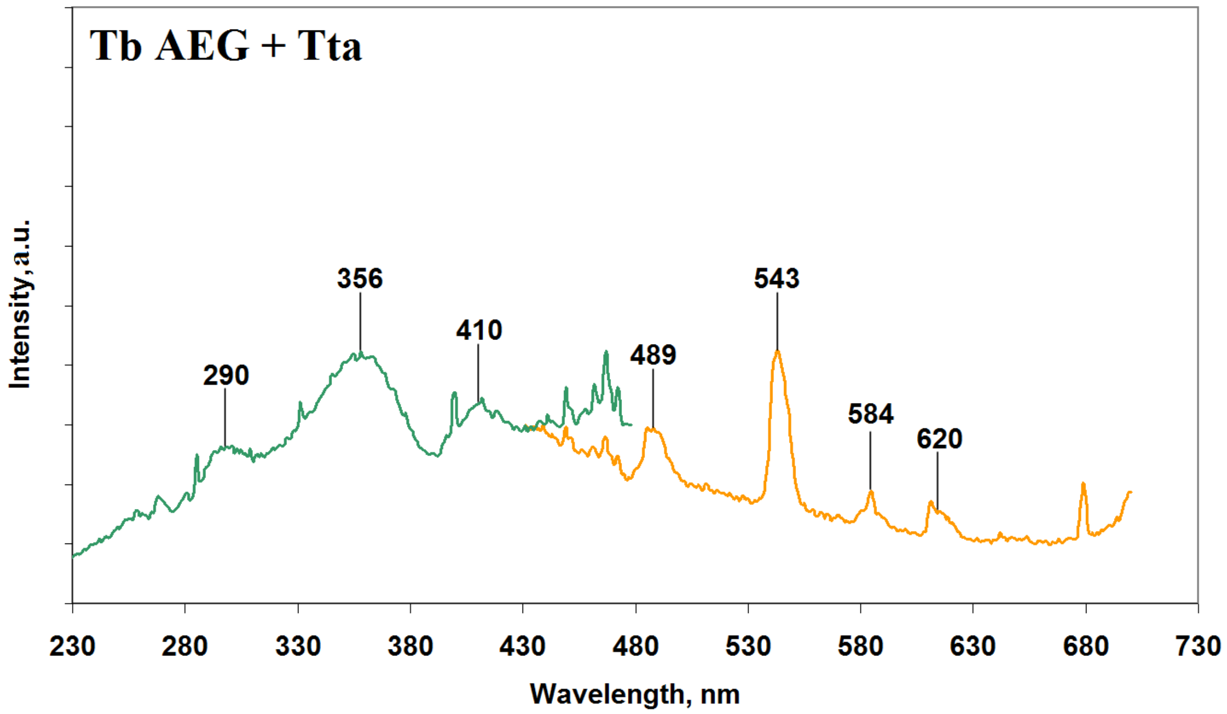


Figure S6. Luminescence (orange curve) and luminescence excitation (green curve) spectra of alginate aerogels crosslinked with $Tb^{3+}$ ions, SC-impregnated with Tta ligands.

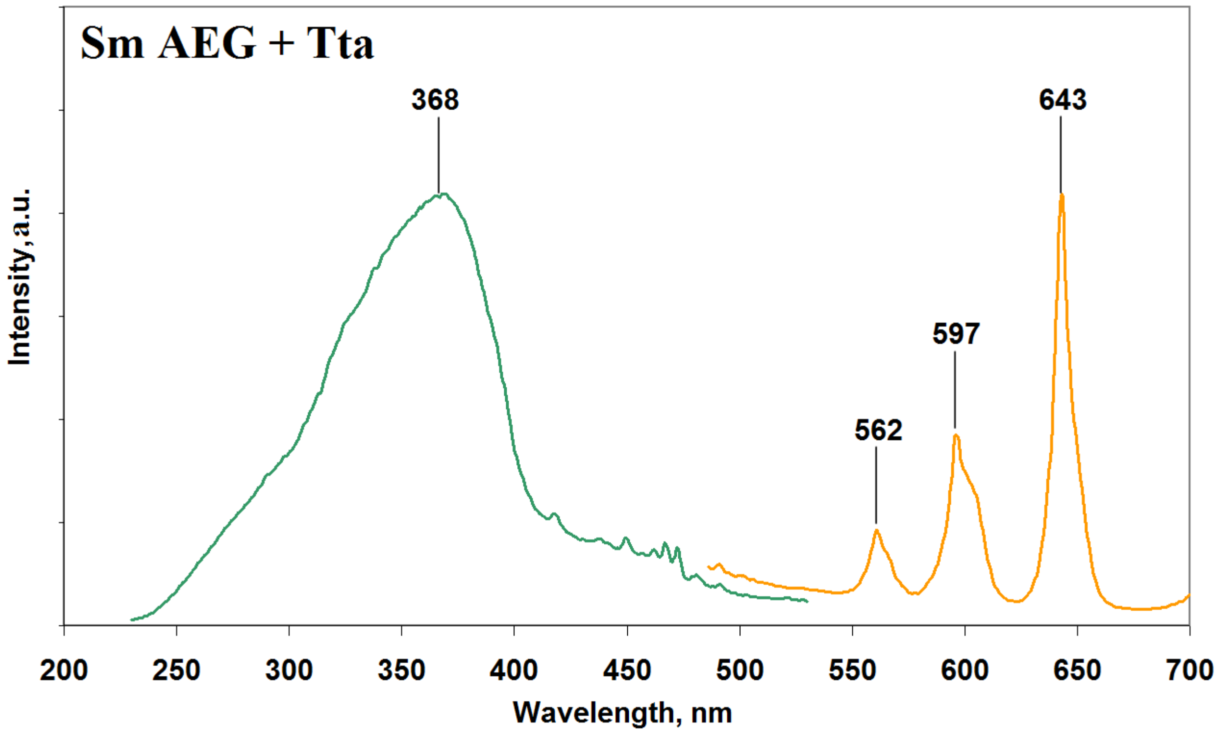


Figure S7. Luminescence (orange curve) and luminescence excitation (green curve) spectra of alginate aerogels crosslinked with $Sm^{3+}$ ions, SC-impregnated with Tta ligands.

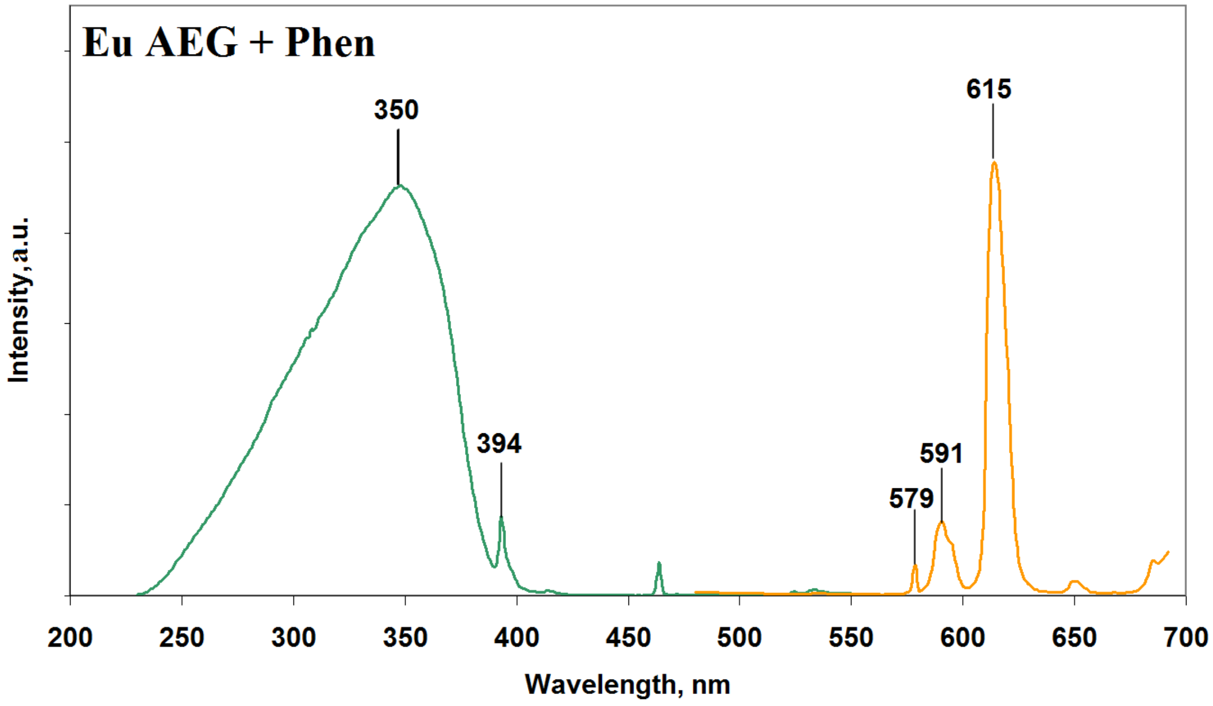


Figure S8. Luminescence (orange curve) and luminescence excitation (green curve) spectra of alginate aerogels crosslinked with $Eu^{3+}$ ions, SC-impregnated with Phen ligands.

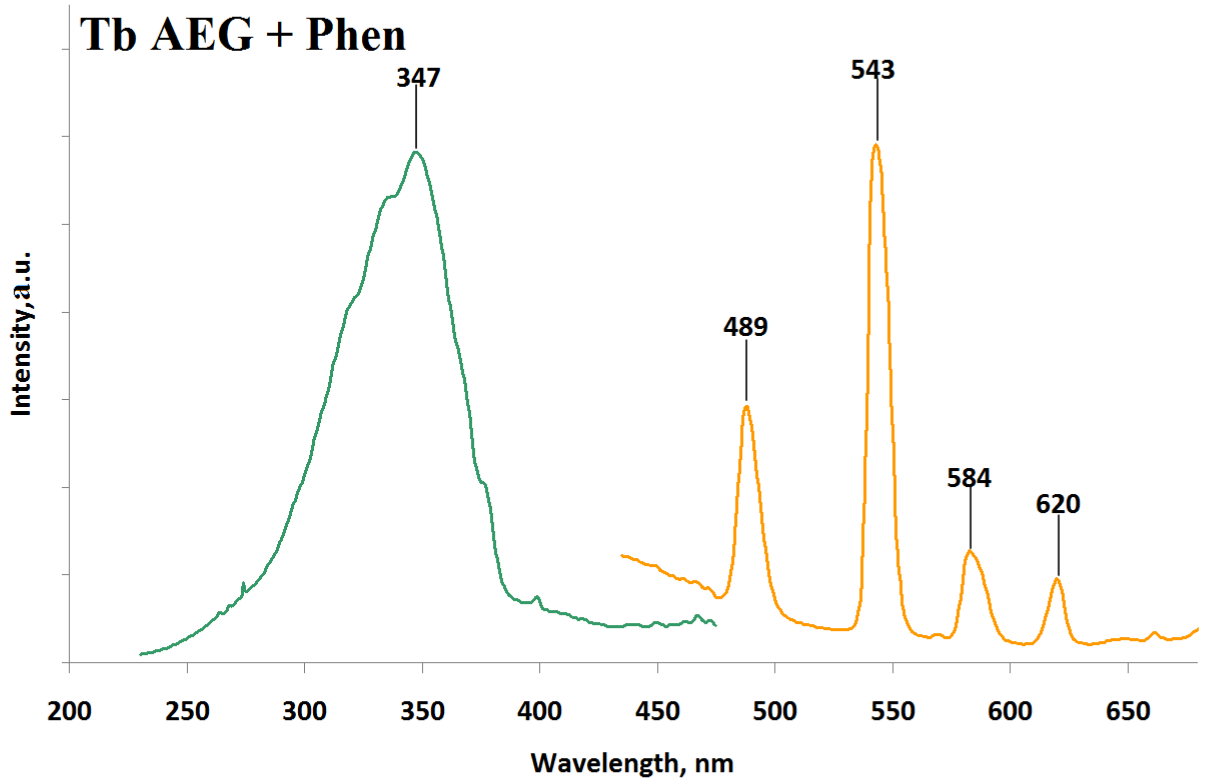


Figure S9. Luminescence (orange curve) and luminescence excitation (green curve) spectra of alginate aerogels crosslinked with $Tb^{3+}$ ions, SC-impregnated with Phen ligands.

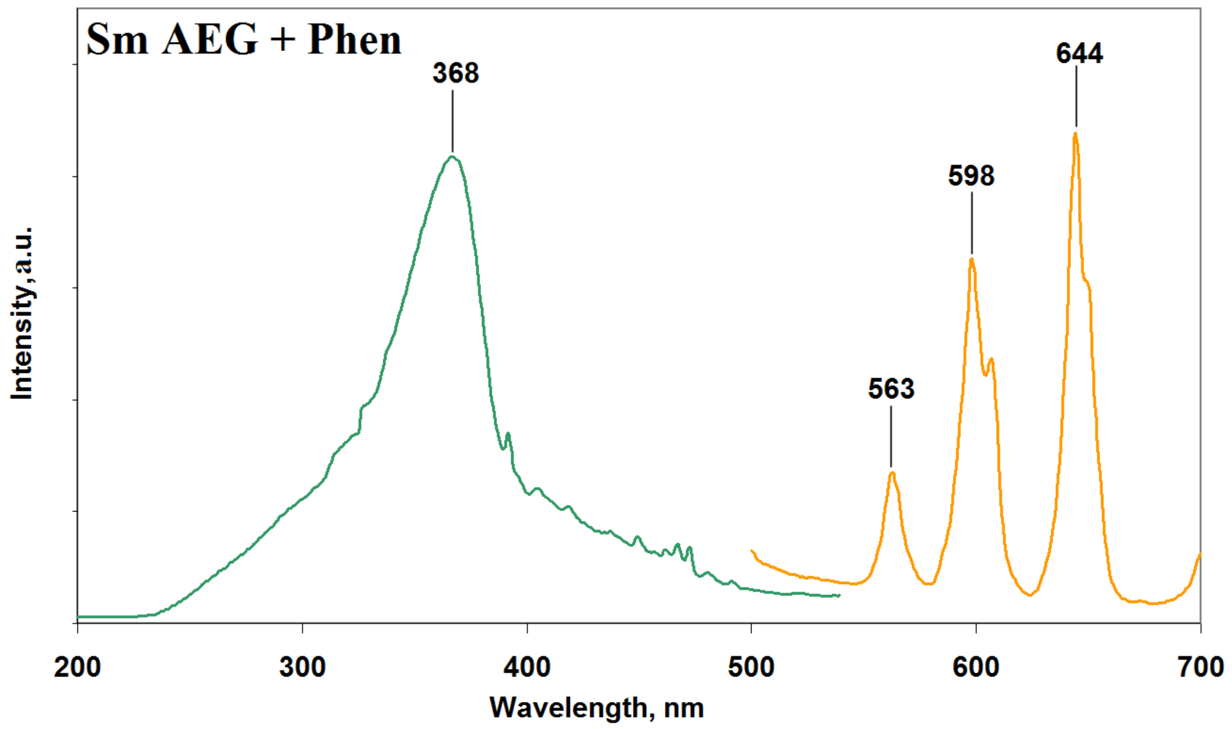


Figure S10. Luminescence (orange curve) and luminescence excitation (green curve) spectra of alginate aerogels crosslinked with $Sm^{3+}$ ions, SC-impregnated with Phen ligands.

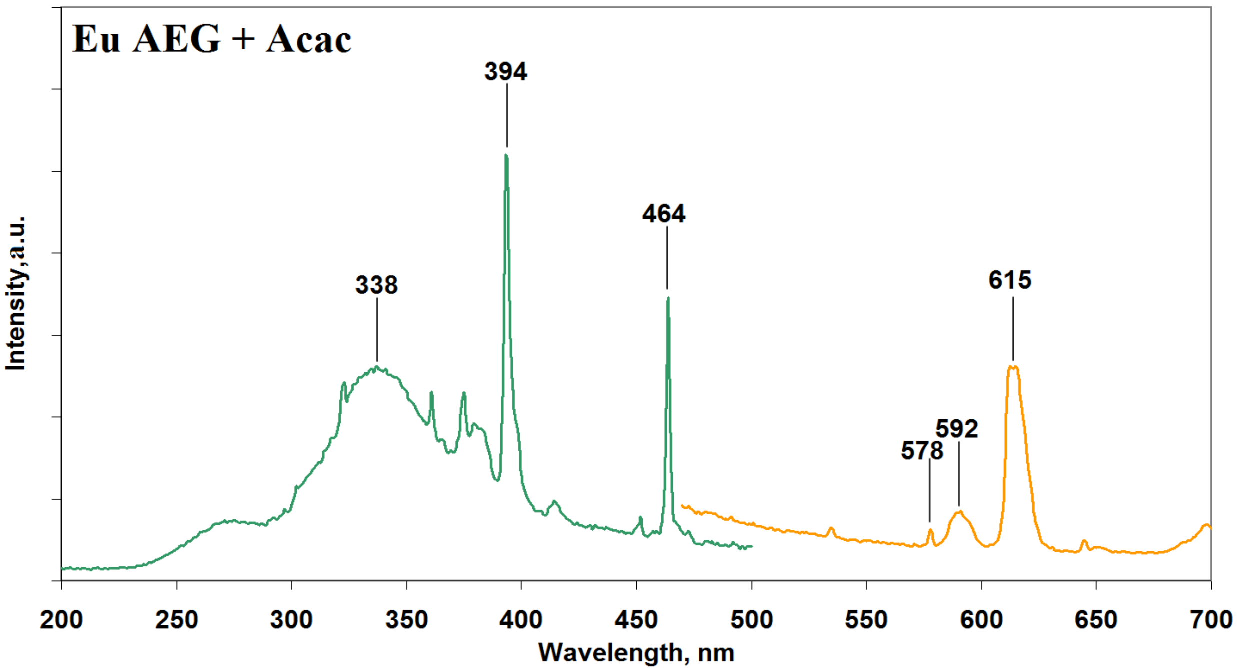


Figure S11. Luminescence (orange curve) and luminescence excitation (green curve) spectra of alginate aerogels crosslinked with $Eu^{3+}$ ions, SC-impregnated with Acac ligands.

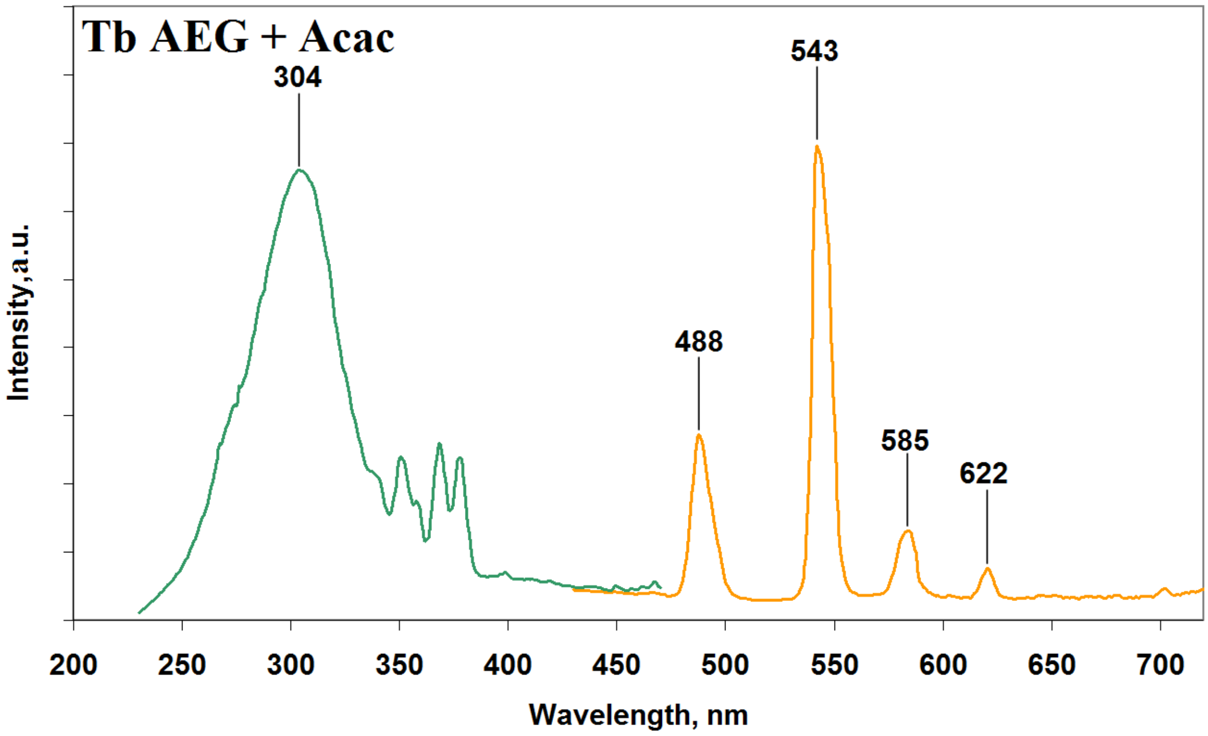


Figure S12. Luminescence (orange curve) and luminescence excitation (green curve) spectra of alginate aerogels crosslinked with $Tb^{3+}$ ions, SC-impregnated with Acac ligands.

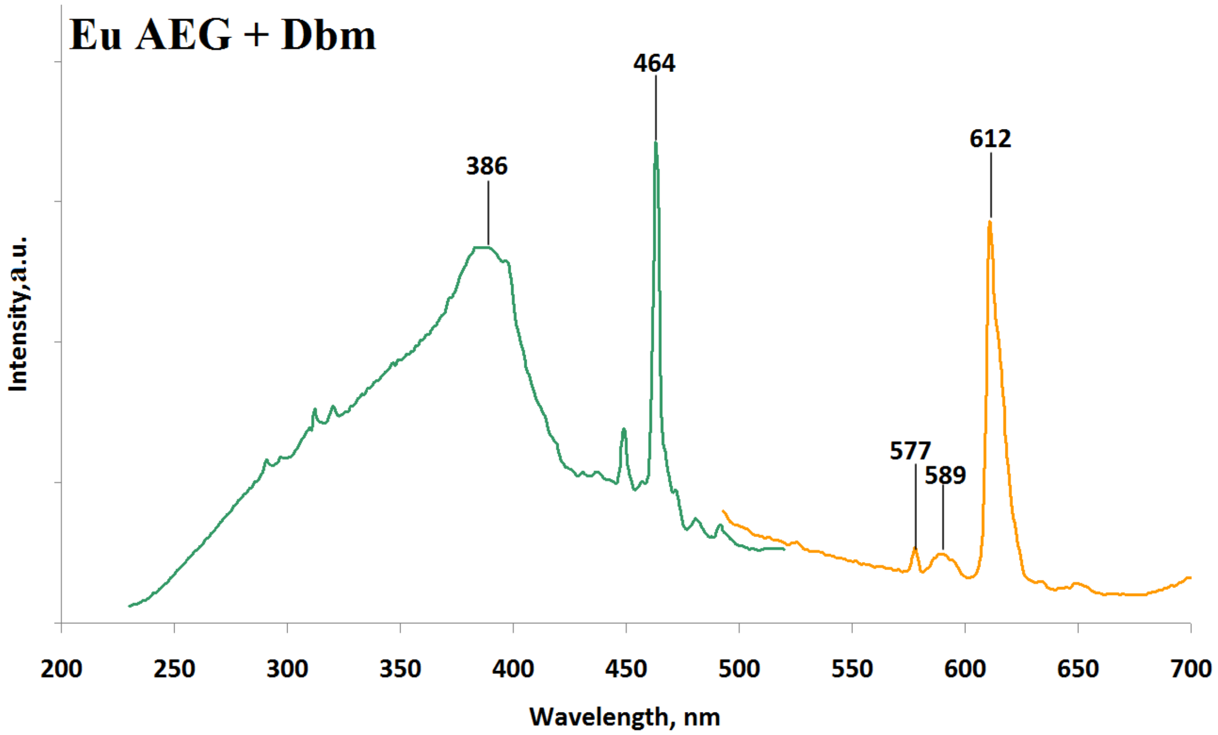


Figure S13. Luminescence (orange curve) and luminescence excitation (green curve) spectra of alginate aerogels crosslinked with $Eu^{3+}$ ions, SC-impregnated with Dbm ligands.

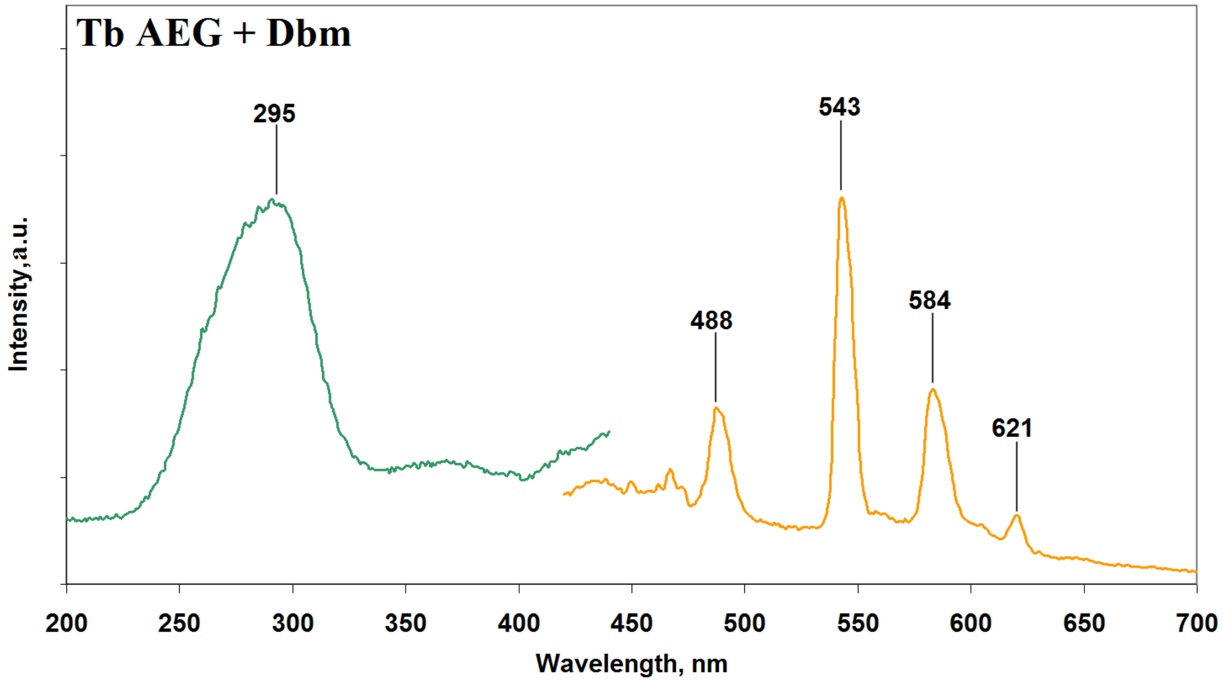


Figure S14. Luminescence (orange curve) and luminescence excitation (green curve) spectra of alginate aerogels crosslinked with $Tb^{3+}$ ions, SC-impregnated with Dbm ligands.

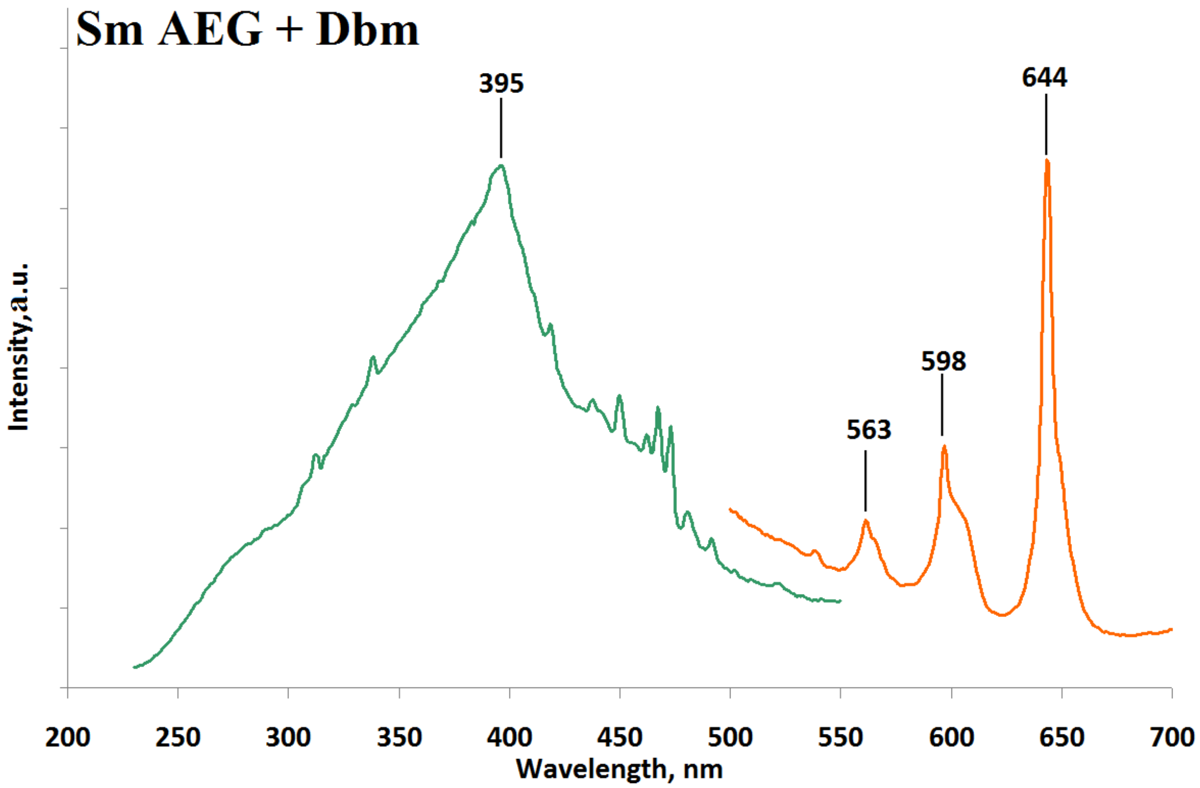


Figure S15. Luminescence (orange curve) and luminescence excitation (green curve) spectra of alginate aerogels crosslinked with $Sm^{3+}$ ions, SC-impregnated with Dbm ligands.

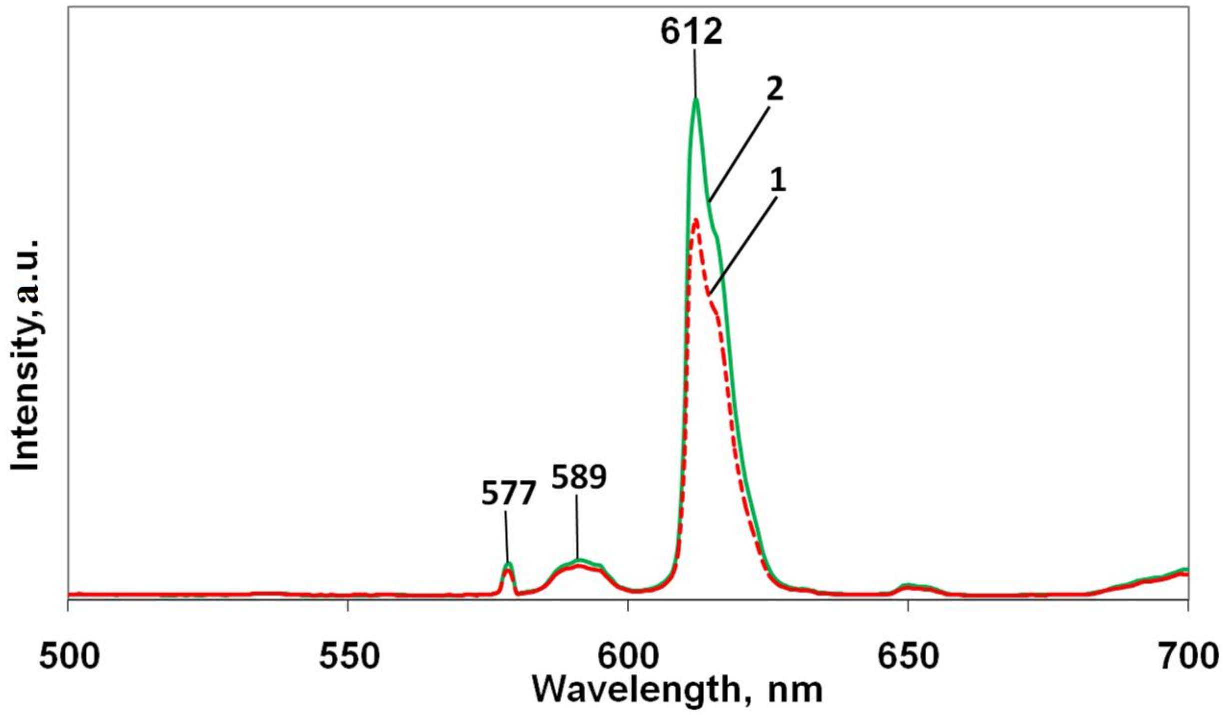


Figure S16. Luminescence spectra: 1 – AEG Eu SC-impregnated with Tta before and 2 – after exposure to acetone vapor.

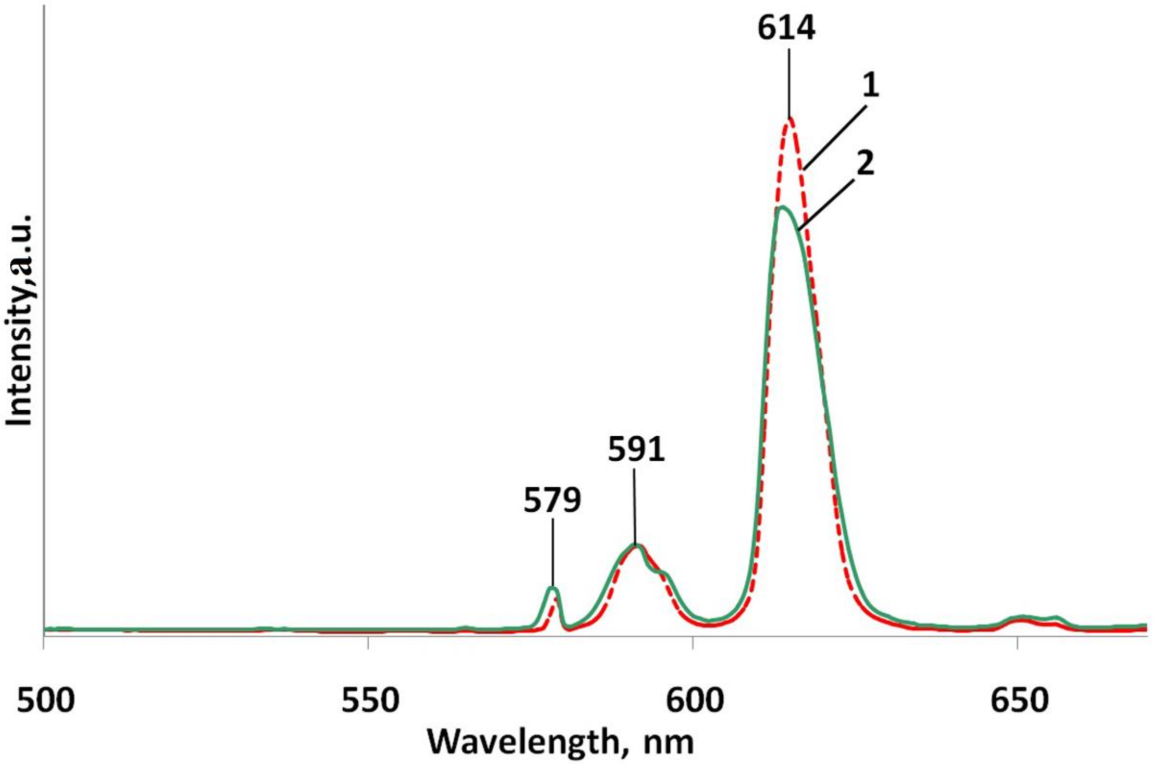


Figure S17. Luminescence spectra: 1 – AEG Eu SC-impregnated with Phen before and 2 – after exposure to ammonia vapor.

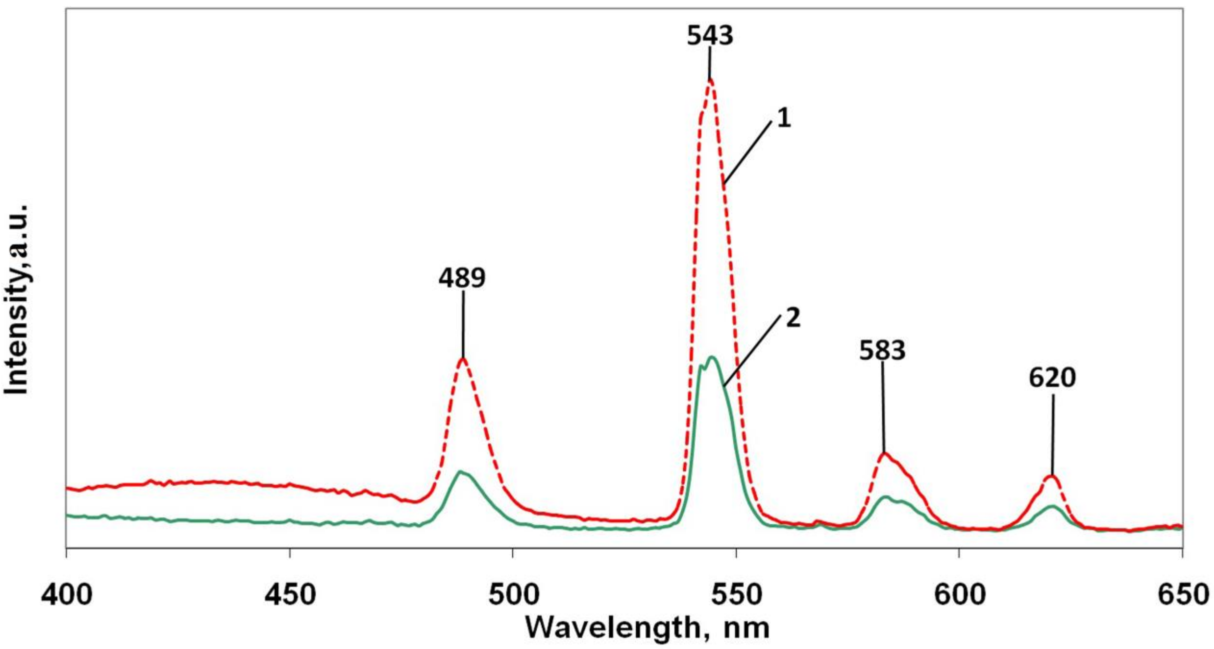


Figure S18. Luminescence spectra: 1 – AEG Tb SC-impregnated with Phen before and 2 – after exposure to ammonia vapor.
